# Induction chemotherapy for the treatment of non-endemic locally advanced nasopharyngeal carcinoma

**DOI:** 10.18632/oncotarget.14279

**Published:** 2016-12-27

**Authors:** Lina Zhao, Man Xu, Wen Jiang, Haitao Pan, Jian Zang, Shanquan Luo, Jianhua Wang, Yongchun Zhou, Mei Shi

**Affiliations:** ^1^ Department of Radiation Oncology, Xijing Hospital, Fourth Military Medical University. Xi’an, China; ^2^ Department of Radiation Oncology, The University of Texas MD Anderson Cancer Center, Houston, TX, 77030, USA; ^3^ Department of Biostatistics, The University of Texas MD Anderson Cancer Center, Houston, TX, 77030, USA

**Keywords:** nasopharyngeal carcinoma, induction chemotherapy, IMRT, non-endemic region, WHO II/III

## Abstract

**Background:**

The role of induction chemotherapy is less clear in non-endemic locally advanced nanopharyngeal carcinomas (NPC).

**Results:**

With a total of 233 eligible patients and a median follow-up of 36 months, 3-year overall survival (OS), local recurrence-free survival (LRFS), distant metastasis-free survival (DMFS), disease free survival (DFS) were 84.5%, 94.9%, 78.6% and 69.2%, respectively. The overall failure rate was 21.0% and distant metastasis occurred in 17.2% patients. Multivariate analyses showed that retropharyngeal and bilateral neck lymph node metastasis were significant prognostic factors for DFS and OS. Moreover, patients receiving both GP (gemcitabine+cisplatin) and TP (docetaxel+cisplatin) regimes had significantly higher DFS and OS compared with PF (cisplatin+5-FU) regime. GP regimes lead to significantly improved OS than TP/PF in some subgroup of patients. No severe toxicities were observed.

**Materials and Methods:**

We retrospectively analyzed stage III-IVb NPC patients treated between Jan 2006 and Dec 2014, with induction chemotherapy followed by concurrent chemoradiation (IC-CCRT). Statistical analyses were performed on survival and failure patterns.

**Conclusions:**

These results suggested IC-CCRT was safe and effective for NPCs from non-endemic region. The choice of induction regimen appeared to affect patient outcomes.

## INTRODUCTION

Nasopharyngeal carcinoma (NPC) is the most common head and neck cancer in Southeast Asia. NPC in endemic areas has different characteristics from those in non-endemic regions likely due to their distinctive pathogenesis. As a result, more than 90% of NPCs in endemic regions exhibit WHO type III histology and had higher detectable EBV DNA levels [1, 2]. Our previously study showed that WHO type II NPC, which represented a higher proportion of cases diagnosed in northwest China (>25%) [3],was a significant factor for poor patient outcomes [4]. Furthermore, less than 15% patients had detectable EBV DNA.

The current standard of care for locally advanced NPC was established by the Intergroup 0099 trial, which recommends concurrent chemoradiation (CCRT) as the preferred treatment option [5]. The introduction of more modern radiation delivery technologies including intensity modulated radiotherapy (IMRT) technique resulted in promising outcomes in NPC patients [6]. However, despite a 5-year local control rate of locally advanced NPC of over 90%, distant metastasis remained the predominant pattern of failure for NPC from both endemic and non-endemic [4, 7, 8], suggesting a need for additional systemic therapy options. Lin *et al* showed that CCRT alone was insufficient for high risk patients [9], and the MAC-NPC meta-analysis demonstrated a significant benefit of adding chemotherapy in the treatment of locally advanced NPC patients [10]. The timing of systemic therapy administration is also not well established, particularly with regard to the role of adjuvant chemotherapy. A phase III trial indicated that adjuvant chemotherapy did not significantly improve 2-year failure-free survival after CCRT in locoregionally advanced NPC [11].

Combining induction chemotherapy and CCRT (IC-CCRT) has attracted more and more attentions. The latest network meta-analysis of the MAC-NPC showed that the regimen with highest probability for being best treatment for DMFS was IC-CCRT (probability 83 %) [10]. Up until now, several randomized studies have reported promising results of IC -CCRT compared with CRT alone [12–17]. However, nearly all studies came from endemic regions. The role of IC-CCRT in IMRT setting for non-endemic NPC with predominantly WHO type II/III histology and lower EBV DNA detectable rate is unclear nor is the best induction chemotherapy regime to be used for these patients. Given the potential distinctive pathogenesis and geographical variations among patients with locally advanced NPCs from non-endemic regions of China, the current study aims to investigate the role of IC-CCRT in the treatment of these patients with WHO II/III NPCs and lower EBV DNA titers and compare the efficacy of different induction regimens.

## RESULTS

### Patients’ characteristics

A total of 233 patients were included in our final analyses. The median age for all patients was 47 years (range 16–74). Median follow-up time was 36 months (range 3–107). All patient and tumor characteristics were summarized in Table 1.

**Table 1:** Patient characteristics and treatment factors for entire series of 233 patients

Characteristic	Patients	
	NO.	%
**Gender**		
Male	171	73.4
Female	62	26.6
**Age (yr)**		
<50y	144	61.8
≥50y	89	38.2
**AJCC**		
T1	24	10.3
T2	63	27.0
T3	53	22.7
T4	93	40.0
**AJCC**		
N0	8	3.4
N1	27	11.6
N2	143	61.4
N3	55	23.6
**Clinical stage**		
III	94	40.3
IVa-b	139	59.7
**Stage group**		
T1-2N2-3	82	35.2
T3-4N0-1	31	13.3
T3-4N2-3	120	51.5
**EB-DNA**		
<5000 copies/ml	220	94.4
≥5000 copies/ml	13	5.6
**Histology**		
WHO II	61	26.2
WHO III	158	67.8
others	14	6.0
**Induction chemotherapy cycles**		
1	22	9.4
2-3	203	87.1
>3	8	3.5
**Induction chemotherapy regimens**		
PF	26	11.2
TP	129	55.4
GP	59	25.3
others	19	8.1
**Retropharyngeal lymph node**		
No	107	45.9
Yes	126	54.1
**Invasion of the skull base**		
No	164	70.4
Yes	69	29.6
**Neck lymph node metastasis**		
None	8	3.4
Unilateral	36	15.5
Bilateral	189	81.1
**Lymph nodes were resected before treatment**		
No	213	91.4
Yes	20	8.6

### Treatment efficacy

**Figure 1:**
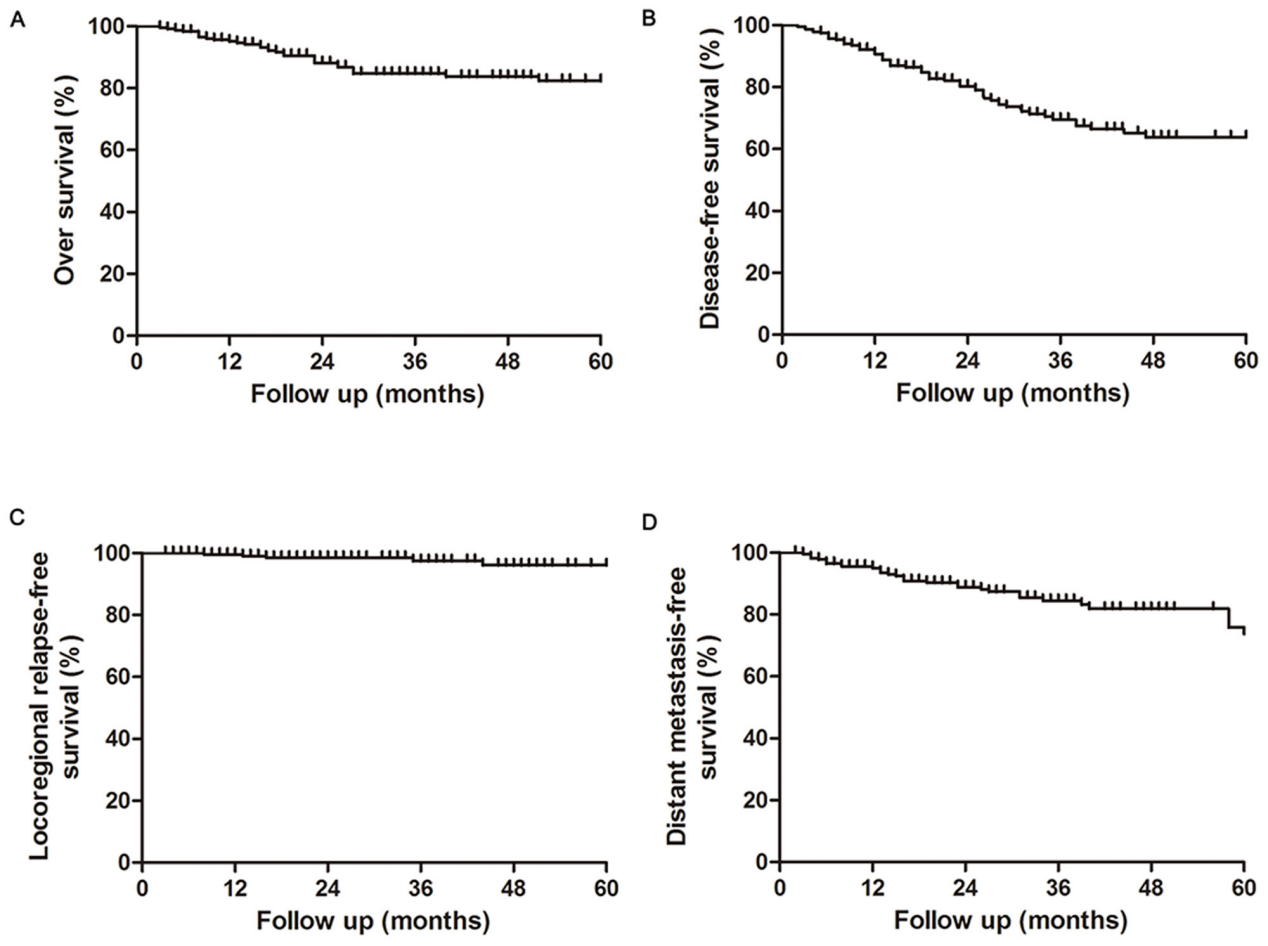
Overall, disease free, localregional relaps-free and distant metastasis-free survival rates in nasopharyngeal carcinoma patients treated with IC-CCRT

Figure 1 showed the survival outcomes for the entire patient cohort. The 3- and 5-year estimated OS, LRFS, DMFS, DFS were 84.5%, 94.9%, 78.6% and 69.2%, and 75.9%, 91.1%, 68.3% and 63.6%, respectively. A total of 200 patients (85.8%) were alive at the conclusion of the study with a median follow-up duration of 35.3 months (range 5-107months). Median OS time was 60.1 months.

### Patterns of failure

**Figure 2:**
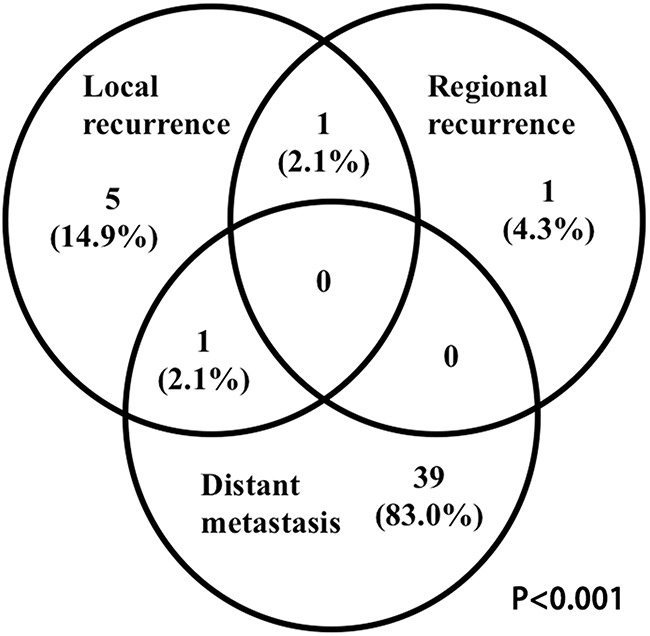
Failure pattern analysis

The overall failure rate for all patients was 21.0% (n=49) (Figure 2). The median time to any recurrence was 16 months (range, 8-23 months). Local and regional recurrence occurred in 7 (3.0%) and 2 patients (0.8%), respectively. The median time to local and regional recurrence was 13.4 months (range: 10-23 months) and 10.0 months (range: 8-10 months), respectively. Among the 9 patients with locoregional recurrences, 7(77.8%) was in-field, 1 (11.1%) was marginal and 1 (11.1%) was out-field. Distant metastasis was noted in 40 patients (17.2%). The median time to distant metastasis was 10 months (range, 2–26 months). The most common metastasis sites were bone (15, 37.5%), lung (9, 22.5%), liver (8, 20.0%) and axillary lymph node (2, 5.0%). Of these patients, 9 (22.5%) metastasized to multiple sites. For the 33 (14.2%) patients who died, causes of death included refractory hemorrhage of nasopharynx (6, 18.2%), tumor related death (26, 78.8%), and unknown causes (1, 3.0%). For the 6 refractory hemorrhage of nasopharynx related death, the causes included tumor recurrence (n=2), radiotherapy complications and nasopharynx mucosa infection during and after radiotherapy (n=2) and tumor re-irradiation related complications (n=2).

### Prognostic analysis

**Figure 3:**
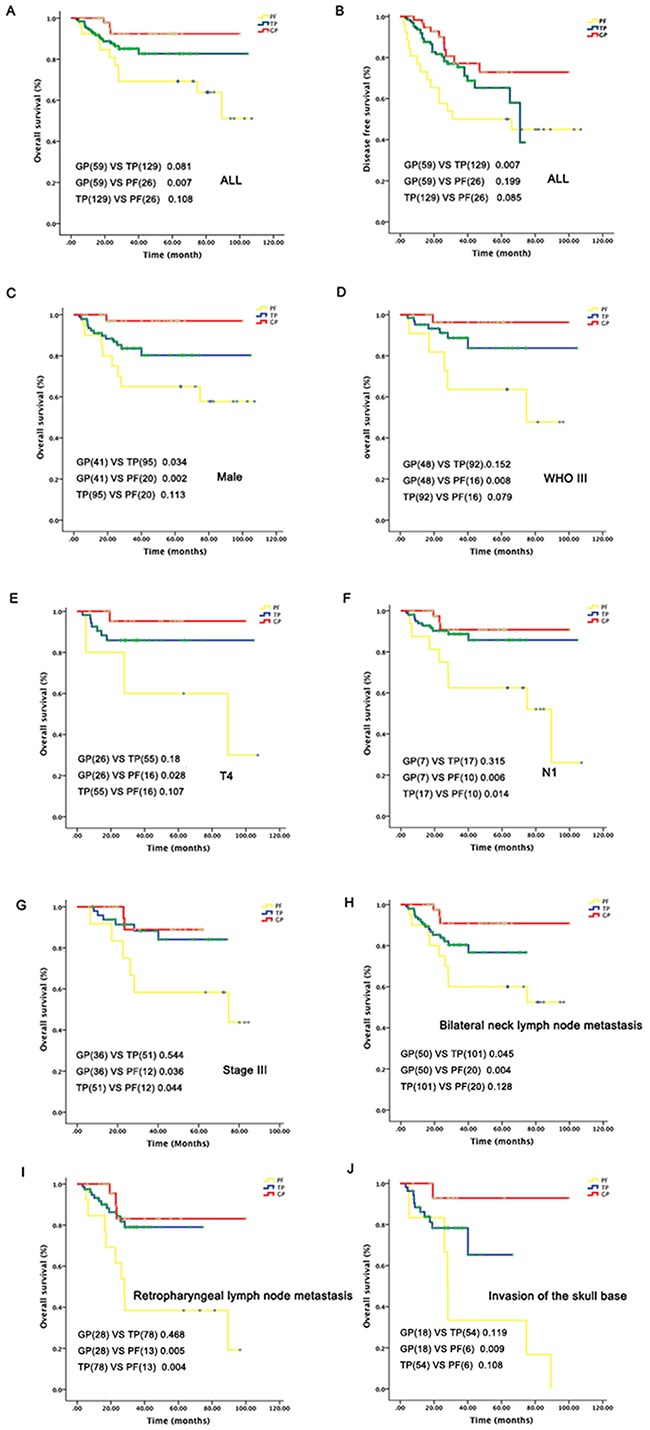
Subgroup analysis of survival outcomes related to different induction chemotherapy regimes

Age, induction chemotherapy regimen (GP vs TP, P=0.081; GP vs PF: P= 0.007, Figure 3A), retropharyngeal lymph node metastasis, invasion of the skull base, bilateral neck lymph node metastasis were found significantly associated with poorer OS in univariate analyses. Gender, induction chemotherapy regimen (GP vs PF: P= 0.007, Figure 3B), retropharyngeal lymph node metastasis, bilateral neck lymph node metastasis was significantly associated with poorer DFS; T3-4N2-3 stage was significantly associated with poorer LRFS (Supplementary Table 1).

Multivariate analyses showed that retropharyngeal lymph node (HR=2.191, 95%CI=1.038-4.625, P=0.024) and bilateral neck lymph node metastasis (HR=3.025, 95%CI=1.277-7.167, P=0.012) were significant prognostic factors for DFS (Table 2). Moreover, patients receiving both GP and TP regime had a significantly higher DFS (GP: HR=0.318, 95% CI= 0.139-0.728, P=0.007;TP: HR=0.514, 95% CI= 0.268-0.987, P=0.046) and OS compared with those received PF regime (GP: HR=0.151, 95% CI= 0.041-0.557, P=0.005; TP: HR=0.371, 95% CI= 0.158-0.873, P=0.023) (Table 2 and Figure 3). Retropharyngeal (HR=2.191, 95% CI=1.038-4.625, P=0.04) or bilateral neck lymph node involvement (HR=12.325, 95% CI=1.536-98.868, P=0.018) was also prognostic for OS (Table 2). No variable was found to be associated with DMFS and LRFS by both uni- and multivariate analyses.

**Table 2:** Multivariate analysis of risk factors for prognosis (n =233)

End point	HR (95% CI)	P-value
**DFS**		
Retropharyngeal lymph node metastasis		
No	1 (reference)	
Yes	1.811(1.041-3.153)	0.036
Neck lymph node metastasis		
Unilateral/none	1 (reference)	
Bilateral	3.025 (1.277-7.167)	0.012
Induction chemotherapy regimes		
PF	1 (reference)	
TP	0.514 (0.268-0.987)	0.046
GP	0.318 (0.139-0.728)	0.007
Gender		NS
**OS**		
Retropharyngeal lymph node metastasis		
No	1 (reference)	
Yes	2.191(1.038-4.625)	0.04
Neck lymph node metastasis		
Unilateral/none	1 (reference)	
Bilateral	12.325 (1.536-98.868)	0.018
Induction chemotherapy regimes		
PF	1 (reference)	
TP	0.371(0.158-0.873)	0.023
GP	0.151 (0.041-0.557)	0.005
Age		NS
Invasion of the skull base		NS

NS, not significant

### Subgroup analysis

Before subgroup analysis, we did Chi-square and/or Fisher's exact test to test whether T stage/N stage/clinical stage and regimes were independent based on the following patient's distribution information. The results showed there were no significant difference in T (p value =0.558), N (X-square=11.004; p value =0.088) and clinical stage (X-square=0.396; p value =0.82) distribution among three treatment groups. Subgroup analyses revealed that GP induction chemotherapy lead to significantly improved OS than PF regime in male patients those with WHO III, T4, N1, and Stage III tumors, or patients with bilateral neck or retropharyngeal lymph node metastasis. Induction chemotherapy with GP also resulted in higher OS compared to TP regime in male and bilateral neck lymph node metastasis patients. For N1, Stage III, and retropharyngeal lymph node metastasis patients, TP resulted in improved OS compared with PF regime (Figure 3C-3J).

### Acute/late toxicity

The toxicities related to different induction chemotherapy regimens were listed in Supplementary Table 2. Acute and late toxicities related to radiation therapy and chemotherapy by site and grade were summarized in Table 3. All patients completed the treatment and were well tolerated, No grade IV toxicities were observed.

**Table 3:** Treatment-related toxicities

Toxicities	No. of patients by toxicity grade (%)				
	0	1	2	3	4
**The acute toxicities**					
Dermatitis	56 (24.0)	94 (40.4)	63 (27.0)	20 (8.6)	0 (0)
Mucositis	60 (25.8)	90 (38.6)	63 (27.0)	20 (8.6)	0 (0)
Dysphagia	47 (20.2)	152 (65.2)	34 (14.6)	0 (0)	0 (0)
Anemia	215 (92.3)	16 (6.9)	2 (0.8)	0 (0)	0 (0)
Thrombocytopenia	206 (88.4)	14 (6.0)	13 (5.6)	0 (0)	0 (0)
Leukopenia	111 (47.6)	75 (32.2)	39 (16.8)	8 (3.4)	0 (0)
Neutropenia	107 (45.9)	63 (27.1)	53 (22.7)	10 (4.3)	0 (0)
Febrile neutropenia	218 (93.6)	14 (6.0)	1 (0.4)	0 (0)	0 (0)
Vomiting	70 (30.0)	140 (60.1)	23 (9.9)	0 (0)	0 (0)
Hand-foot syndrome	0 (0)	0 (0)	0 (0)	0 (0)	0 (0)
Ototoxicity	197 (84.5)	36 (15.5)	0 (0)	0 (0)	0 (0)
Diarrhea	230 (98.7)	3 (1.3)	0 (0)	0 (0)	0 (0)
Hepatoxicity	215 (92.3)	15 (6.4)	3 (1.3)	0 (0)	0 (0)
Nephrotoxicity	212 (91.0)	19 (8.2)	2 (0.8)	0 (0)	0 (0)
Neuropathy	0 (0)	0 (0)	0 (0)	0 (0)	0 (0)
**The late toxicities**					
Xerostomia	53 (22.7)	34 (14.6)	112 (48.1)	34 (14.6)	0 (0)
Neck fibrosis	227 (97.4)	6 (2.6)	0 (0)	0 (0)	0 (0)
Trismus	228 (97.9)	5 (2.1)	0 (0)	0 (0)	0 (0)
Dysphagia	213 (91.4)	14 (6.0)	6 (2.6)	0 (0)	0 (0)
Hearing impairment	63 (27.1)	166 (71.2)	4 (1.7)	0 (0)	0 (0)
Temporal necrosis	0 (0)	0 (0)	0 (0)	0 (0)	0 (0)
Cranial nerve palsy	231 (99.1)	2 (0.9)	0 (0)	0 (0)	0 (0)

The primary radiation-related acute toxicities were dermatitis, mucositis and dysphagia, which were generally mild or moderate. The worst acute toxicity was grade 3 dermatitis in 8.6% patients, grade 3 mucositis in 8.6% patients and grade 2 dysphagia in 14.6% patients. All patients were able to complete the whole course of irradiation without treatment interruption. The most common chemotherapy related toxicities were vomiting (60.1% grade 1) and neutropenia (27.1% grade 1). Grade 2 vomiting was observed in 9.9% patients and grade 3 neutropenia in 4.3% patients.

In terms of late toxicities, xerostomia, neck fibrosis, trismus, dysphagia, hearing impairment, temporal necrosis and cranial nerve palsy were noted in our patients. The most common grade 1 late toxicities were hearing impairment and xerostomia, accounting for 71.2% and 14.6% patients, respectively. Grade 2 hearing impairment and xerostomia occurred in 1.7% and 48.1% patients, respectively.

## DISCUSSION

The need for improved distant metastasis control has promoted increased re-exploration of systemic therapies in the treatment of locally advanced NPC. Induction chemotherapy is especially suitable for bulky primary lesions and/or extensive nodal disease, and might help to treat those NPCs with higher potential for metastasis [18]. With the significant improvement in local disease control in the era of IMRT, distant metastasis is often the predominant failure pattern [4, 19].

The use of IC-CCRT in the treatment of locally advanced NPCs have yielded promising results in the modern era in combination with IMRT with a reported 5-year OS rate of 78% [20] and 84% [19] in two recent studies. Currently, at least three phase III trials (NCT01245959, NCT00201396 and NCT00379262) are conducting investigations into whether patients with advanced NPC may benefit more from IC-CCRT. However, majority of the studies emphasize on NPC patients from endemic regions, with scarcity of data focus non-endemic NPCs. Our study results adds to the limited literature on non-endemic NPCs patients treated with IC-CCRT. Again, we demonstrated that while local control rate was satisfactory, distant metastasis remain the majority cause of failure in 17.2% patients.

The survival outcomes of our patient cohort is slightly worse than previously reported NPC patients from endemic regions. Tumor biology and pathology may play an important role in determining this differences. Although more than 90% of NPCs in endemic regions belong to WHO type III [2], our previously study demonstrated that WHO type II was the predominant histology in northwest China and was independent predictors for DMFS, LRFS and OS [4, 21]. In our present study, 26.2% of the patients had WHO type II histology, which was confirmed by experienced pathologists from endemic regions of China. This proportion is higher than reported in endemic regions. Although WHO type II status alone was not an independent predictor of survival in the current study, subgroup analysis showed that GP induction chemotherapy regime was significantly associated with better OS in WHO type III patients, indicating potential benefit of IC+CCRT in this subpopulation of patients.

The role of EBV driven pathogenesis in NPC is well established, and EBV positivity in NPC patients is an independent predictor for patient outcomes [22]. In endemic regions of China, EBV DNA was detected in nearly 90% NPC patients [1], which is significantly higher compared to the 10% detection rate in the present study and 15.9% in our previous study [7]. Guo et al. preformed a matched analysis and found that the IC+CCRT provide significant benefit in very-high risk patients (stage N2-3 with EBV DNA >/=4000 copies/ml), with a reported 5-year OS of 84.3% versus 67.5% in patients treated with concurrent chemoradiation alone (P =0.033) [23]. Due to the relative small number of patients with EBV DNA positivity, we could not observe significant difference in survival outcomes, stressing the importance of other clinicopathological features that determine patient outcomes.

The question of optimal induction chemotherapy regimen remains unclear. Classical combination of cisplatin and fluorouracil has been reported to be effective and widely used in locoregionally advanced NPC. However, they tend to increase the risks of serious mucositis combined with RT, indicating more ideal regimens are needed. Other chemotherapeutic agents including docetaxel and gemcitabine have been incorporated in the treatment of NPC. Multiple studies have suggested that induction with TP or GP regimen are suited for the treatment of advanced NPC. Hui etal performed a randomized trial and found TP induction regime improved NPC survival [14]. Lim etal found that carboplatin and gemcitabine is a promising IC regimen for the treatment of locally advanced NPC, with 3 year OS rate of 89.3% [24]. Direct comparison of different induction chemotherapies has also been carried out. Ou etal found TPF/TP and GP showed a trend of improving 5-year survival and they recommended taxane and gemcitabine-comprising regimen [19]. Another report indicated GP regimen may be superior to TP/FP regimen in treating locoregionally advanced NPC in terms of better OS and a trend toward better DMFS [25]. Tianet.al suggested that taxane-containing IC regimens may be more efficient for short-term local control in Chinese patients with locally advanced NPC than the non-taxane-containing regimens [26]. Despite these evidences, a major concern is that majority of the studies were carried out on endemic NPC patients. Our study focused on different patient populations from non-endemic region of China and found that these patients receiving both GP and TP regime had a significantly higher DFS and OS rate compared with those received PF regime, and GP had a trend towards better survival than TP regime.

Some reports also investigated the special subgroup of patients who could benefit from some IC regimes. One study showed that among patients with T4N1-2M0 and stage IVb, taxanes-based IC significantly improved the 4-year DMFS by 11.2% and marginally improved FFS and OS [27]. This study also indicated male and bilateral neck lymph node metastasis patients might benefit from GP regimes compared with TP and PF regimes.

Because of its retrospective nature, our study has several limitations including the potential confounding factors as well as limited patient numbers that may affect the final conclusion of the study. In addition, the single institutional nature of the study may also limit the applicability of our findings for patients from other geographical regions and institutions.

In conclusion, our experience suggested IC-CCRT in the treatment of NPC was safe and effective in non-endemic regions. GP had a trend towards better survival than TP regime and GP/ TP regime had a significantly better DFS and OS compared than PF regime. This study indicated a trend to changing from PF IC regime to GP/TP regime. Further validation of our findings would be expected.

## MATERIALS AND METHODS

### Patients characteristics

All patients with newly diagnosed and histological proven stage III-IVb (AJCC 2002) non-keratinizing NPCs treated at the department of radiation oncology in Xijing Hospital, Fourth Military Medical University between Jan 2006 and Dec 2014 were screened for the study. Only patients from northwest region of China were included. Other eligibility criteria include Karnofsky performance score≥70 and no evidence of distant metastasis. Histological grading was done according to the 2003 World Health Organization (WHO) NPC classification criteria. This study had been approved by ethics committee of Xijing hospital. Plasma EBV DNA sample was collected and fluorescence polymerase chain reaction (PCR) was performed by using EBV PCR quantitative diagnostic kit (Da-An Genetic Diagnostic Center, Guangzhou, China).

### Radiotherapy

All patients were treated with IMRT within 14-20 days after neoadjuvant chemotherapy. IMRT was delivered with a simultaneous-integrated boost (SIB) technique. The gross tumor volume (GTV) included the nasopharynx gross tumor volume (GTVnx) and positive neck lymph nodes (GTVnd), which were delineated based on post-and pre-chemotherapy images, respectively. The high-risk clinical tumor volume (CTV1) expands from the GTV and included the entire nasopharyngeal mucosa, retropharyngeal lymph nodes, skull base, parapharyngeal space, pterygopalatine fossa, sphenoid sinus, posterior third of the nasal cavity and maxillary sinus. The low-risk clinical tumor volume (CTV2) included those without lymph node metastasis covering the lower neck and supraclavicular fossa. The planning target volume (PTV) was created with a 3-mm margin from the GTV and CTV, respectively, to account for daily set-up errors during treatment. The prescribed doses were 70–74 Gy to the PTV for gross primary disease, and 68–74 Gy for positive lymph nodes in 30-33 fractions; the prescribed doses for high risk and low risk region PTV were 60-64 Gy in 33 fractions and 50-54 Gy in 30-33 fractions, respectively. The doses received by the organs at risk were limited below tolerance levels [28].

### Chemotherapy

All patients received neoadjuvant chemotherapies consisted of 1-4 cycles of PF (cisplatin 30 mg/m^2^/d IV for 3 days, 5-FU 800-1000 mg/m^2^ IV on d1-5),TP (Docetaxel 75 mg/m^2^ IV ond1, cisplatin 30 mg/m^2^/ d IV for 3 days) or GP regimen (gemcitabine1000 mg/m2 IV on d1, d8, cisplatin 30 mg/m2/ d IV for 3 days). Induction chemotherapy regimens were chosen according to physician's preference or based on the criteria of clinical Trial we have participated in. Chemotherapies were typically given 2-3 weeks prior to the initiation of CCRT. The concurrent chemotherapy consisted cisplatin at 80-100mg/m2 on days 1-3 at 3 week interval. No patient received adjuvant chemotherapy. Blood tests for liver and renal functions performed during the entire chemotherapy course.

### Follow-up

Patients were assessed at regular intervals for treatment response and toxicity, both during (weekly) and after radiation therapy (every 2–3 months during the first 2 years, then every 3–4 months during years 3–5, and annually thereafter). Flexible nasoendoscopy was performed at every visit. Magnetic resonance imaging (MRI) of the head and neck was performed every 3 to 6 months in the first 3 years. Chest computerized tomography (CT), abdominal CT or sonography and bone scan were done at least every year or when clinically indicated to detect recurrence or metastasis.

### Acute and late toxicities

Radiotherapy-related toxicities were evaluated and scored on a weekly basis according to the Acute and the Late Radiation Morbidity Scoring Criteria of RTOG. Chemotherapy-related toxicities were graded by the WHO criteria. Radiotherapy-related late toxicities were reported in patients whose follow-up period was over 1 year.

### Statistical analysis

Treatment failures were classified as ‘in-field’, ‘marginal’ or ‘out-field’ if at least >95%, =20–95% or <20% of the volume of failure were within the 95% isodose line of the high risk PTV prescription dose, respectively [29]. Overall survival (OS), distant metastasis-free survival (DMFS), freedom locoregional relapse-free survival (LRFS), and disease-free survival (DFS) rates were calculated using Kaplan–Meier analysis and compared with log-rank test. OS was defined as the time from the first day of treatment to the date of death. DMFS was defined as the time from the first date of treatment until the date of distant failure and LRFS was defined as the time from the first date of treatment until the date of localregional failure. DFS was defined as the time of treatment to an event (local or distant relapse or death). Sub-group analyses were based on Kaplan-Meier analysis. Univariate analysis and multivariate analysis were conducted by using Cox proportional hazards model. Factors with p values less than 0.10 by univariate analyses were then entered into multivariate Cox proportional hazards regression analysis with backward stepwise variable selection. Final fitted models included all significant factors with p<0.05. All analyses were performed by using SPSS 19.0 for Windows (SPSS, Chicago, IL).

## SUPPLEMENTARY MATERIALS TABLES






